# Risk of Mortality Prediction Involving Time-Varying Covariates for Patients with Heart Failure Using Deep Learning

**DOI:** 10.3390/diagnostics12122947

**Published:** 2022-11-25

**Authors:** Keijiro Nakamura, Xue Zhou, Naohiko Sahara, Yasutake Toyoda, Yoshinari Enomoto, Hidehiko Hara, Mahito Noro, Kaoru Sugi, Ming Huang, Masao Moroi, Masato Nakamura, Xin Zhu

**Affiliations:** 1 Division of Cardiovascular Medicine, Toho University Ohashi Medical Center, Tokyo 153-8515, Japan; 2 Graduate School of Science and Technology, Nara Institute of Science and Technology, Ikoma 630-0192, Japan; 3 Division of Cardiovascular Medicine, Odawara Cardiovascular Hospital, Odawara 250-0873, Japan; 4 Graduate Department of Computer and Information Systems, The University of Aizu, Aizuwakamatsu 965-8580, Japan

**Keywords:** deep learning, heart failure, mortality, risk prediction, time-varying covariates

## Abstract

Heart failure (HF) is challenging public medical and healthcare systems. This study aimed to develop and validate a novel deep learning-based prognostic model to predict the risk of all-cause mortality for patients with HF. We also compared the performance of the proposed model with those of classical deep learning- and traditional statistical-based models. The present study enrolled 730 patients with HF hospitalized at Toho University Ohashi Medical Center between April 2016 and March 2020. A recurrent neural network-based model (RNNSurv) involving time-varying covariates was developed and validated. The proposed RNNSurv showed better prediction performance than those of a deep feed-forward neural network-based model (referred as “DeepSurv”) and a multivariate Cox proportional hazard model in view of discrimination (C-index: 0.839 vs. 0.755 vs. 0.762, respectively), calibration (better fit with a 45-degree line), and ability of risk stratification, especially identifying patients with high risk of mortality. The proposed RNNSurv demonstrated an improved prediction performance in consideration of temporal information from time-varying covariates that could assist clinical decision-making. Additionally, this study found that significant risk and protective factors of mortality were specific to risk levels, highlighting the demand for an individual-specific clinical strategy instead of a uniform one for all patients.

## 1. Introduction

Heart failure (HF) has been significantly associated with mortality, especially among those older than 65 years [1,2]. Japan has a “super-aged” society, and the number of patients with HF was estimated to reach 1.3 million by 2030 [3]. Although a better survival prognosis in Japan than that in Europe had been observed, the length of hospital stays in Japan was approximately three times that in western countries [4]. Simultaneously, HF was a leading cause of hospitalization in Japan, bringing a heavy economic burden to society [5]. Considering these facts, a prognostic prediction model is expected to inform patients and their families about the course of the disease and guide physicians to more optimal treatment and management strategies such as allocation of medical resources in consideration of survival prognosis.

The multivariate Cox proportional hazard (CPH) model [6] is the most frequently used statistical-based method for risk prediction, but its performance is somewhat limited by the linearity of the functional form assumed in the partial hazard (exponential) part [7,8]. By contrast, deep learning-based prediction models can fit and learn more complicated (e.g., non-linearity by activation function) relationships between covariates and outcomes. A fully connected (FC) neural network-based survival prediction model, referred to “DeepSurv”, has been proposed by Katzman et al. [9]. It achieved better prediction performances than the CPH model for cancer survival [10] and cardiovascular risk [8], and displayed a workable performance on early triage of critically ill patients with COVID-19 [11].

Despite the widespread use of the CPH model and performance improvement achieved by DeepSurv, the two models only consider invariant covariates but have no ability to learn from time-varying covariates. Although a time-varying CPH model has been proposed to take time-varying covariates into consideration [12], this model deals with the time-dependent issue by simply considering the hazard at time t depending on the value of the time-varying covariate at that time but the regression effect or the weight of the covariate being constant or invariant. In essence, it is similar to a traditional CPH model. Therefore, the model cannot capture the temporal relationship implied in time-varying covariates. Furthermore, the time-varying CPH model requires the dataset to be pre-processed into a so-called “long” format, where each duration is represented in a start and stop view, which is monotonous. In this study, a recurrent neural network (RNN)-based model, referred to as “RNNSurv”, was developed and temporally validated. Temporal validation (or narrow validation) was defined by Moons et al. as follows: “External validation may use participant data collected by the same investigators, typically using the same predictor and outcome definitions and measurements, but sampled from a later period” [13]. The proposed model could learn temporal features from time-varying covariates and fit nonlinear relationship to predict risk of all-cause mortality for patients with HF. RNNSurv demonstrated better performance than CPH and DeepSurv in terms of discrimination, calibration, and ability of risk stratification, especially identifying the patients with high risk of mortality. Patients’ characteristics varied by risk level in view of the stratified risk groups (high risk and other (or low risk)). This study also suggested that significant risk and protective factors of mortality were required to be discussed specific to risk levels.

## 2. Materials and Methods

### 2.1. Study Design and Data Collection

The protocol for this retrospective study was prepared in accordance with the Declaration of Helsinki, and the study was approved by the Institutional Review Board and Ethics Committee of Ohashi Medical Center, School of Medicine, Toho University (No. H19031).

The data collection was the same as that explained in a previous study [14]. Medical records of patients with HF hospitalized at Toho University Ohashi Medical Center between April 2016 and March 2019 were reviewed by cardiovascular specialists, constituting a development dataset. Then, an independent validation dataset was built from patients admitted during the period of one year later. HF was diagnosed based on the following information in the medical records: clinical syndromes consisting of dyspnea, malaise, edema, or decreased exercise capacity due to the loss of compensation of cardiac pump function, secondary to structural or functional abnormalities of the heart [15].

Patient characteristics, laboratory data, and echocardiographic data were manually extracted from electronic medical records by a hired data collection staff. In addition, diagnosis procedure combination (DPC) data were also added to build a high-quality database in this study. DPC is made up of unified patient clinical information in Japan, patient information including disease name, surgical procedure, various stage classifications, medical expenses, and patients’ medical practice is converted into electronic data, and medical insurance services such as claims are provided in Japan based on this DPC [16]. After verifying consistency between DPC data and medical records data, the database for this study was created. The database construction flow is shown in Supplementary Figure S1 and details of patients information are summarized in Supplementary Table S1.

**Figure 1:**
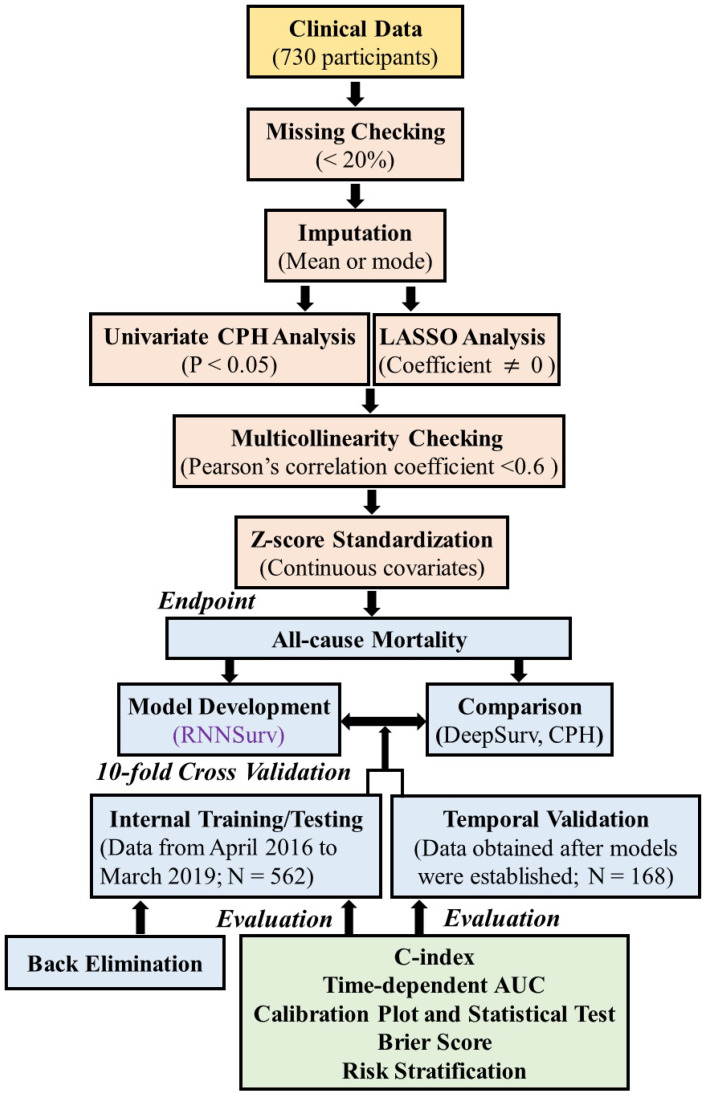
Overview of the proposed prognostic prediction system.

Figure 1 shows the flowchart of the proposed prognostic prediction system. It mainly consists of predictors’ selection, model development and validation, evaluation from discrimination, calibration, overall aspects [17], and risk stratification.

### 2.2. Outcome Definition

The endpoint was evaluated as all-cause mortality including both cardiovascular and non-cardiovascular mortality. Cardiovascular mortality was defined as death due to acute myocardial infarction, sudden cardiac death, HF, stroke, cardiovascular procedures, cardiovascular hemorrhage, and other cardiovascular deaths that could not be attributed to non-cardiac causes. Non-cardiovascular mortality was defined as any death that was not thought to be the result of a cardiovascular cause, such as infection and/or malignancy.

### 2.3. Preprocessing and Statistical Analysis

Candidate predictors were identified by preprocessing and statistical analysis, as previously described [18]. First, covariates that were missing in more than 20% of patients were excluded. As multiple imputation needs to be trained and is hard to converge with a relatively small sample size but a high dimension of variables, in addition, after checking the missing distribution, the missingness distribution seems balanced between death and survival groups as illustrated in Supplementary Table S2, and the continuous and categorical covariates with missing data were imputed using the simple imputation method [19]. Then, covariates significantly associated with mortality were identified by the univariate CPH model and least absolute shrinkage and selection operator regression [20] together. Multicollinearity was assessed and excluded by Pearson’s correlation coefficient between pairs of continuous variables (setting 0.60 as a threshold) as was performed by Mesquita et al. [21]. Thereafter, the remaining covariates were identified as candidate predictors, where continuous predictors were Z-score standardized [22] when inputting to models. Available covariates were presented as mean ± standard deviation or frequency (percentage) in view of the covariates’ type. For group comparison, a two-tailed, unpaired Student’s *t*-test was used to assess the difference of continuous covariates with normal distribution, and a Mann–Whitney test was employed for skewed continuous covariates, where normality was tested using the Shapiro–Wilk test. Categorical data were compared using Pearson’s Chi-square test or Fisher’s exact test, as appropriate. A *p* value < 0.05 was considered statistically significant. Data preprocessing was performed using Python (Python Software Foundation, Beaverton, OR, USA; version 3.7.7), and statistical analysis was conducted using R (R Foundation for Statistical Computing, Vienna, Austria; version x64 3.6.0).

### 2.4. Model Development and Validation

**Figure 2:**
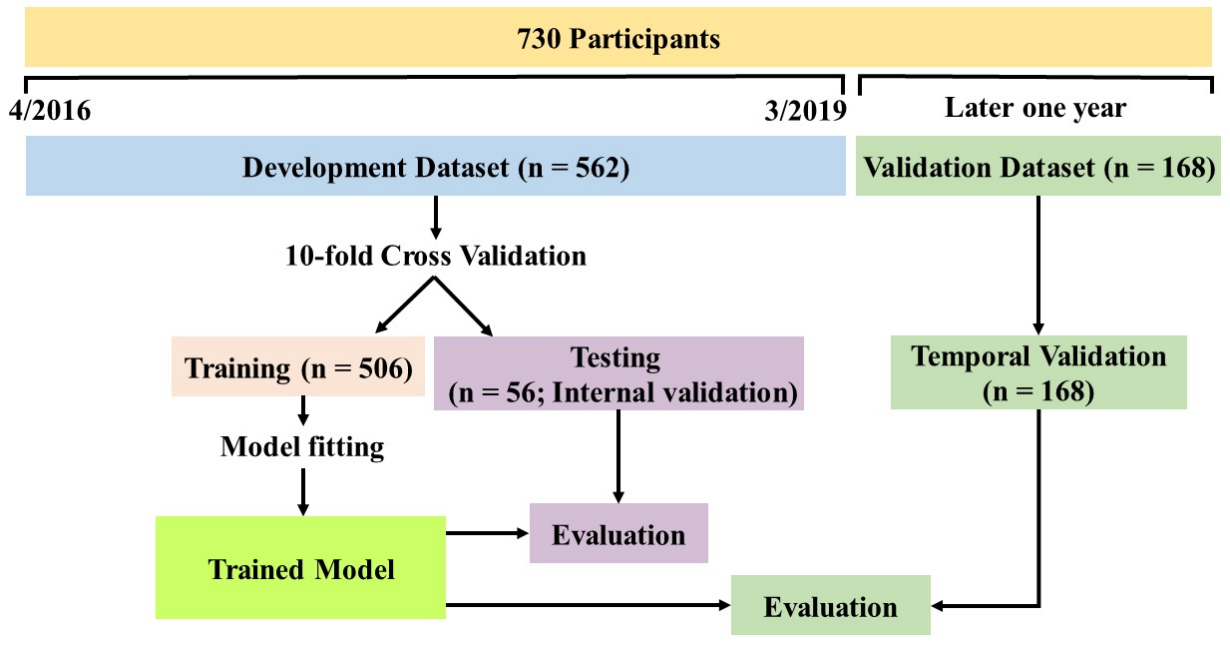
Overview of model development and validation.

The proposed RNNSurv’s input includes two categories of candidate predictors: invariant (collected once) and time-varying (collected at both admission and discharge) with different input strategies. RNNSurv consists of two branches as shown in Supplementary Figure S2. The first is an RNN layer with inputs of time-step dynamic predictors (at admission and discharge). The second branch is an FC layer with inputs of invariant predictors. Each FC layer connects with a ReLu activation function and batch normalization, and is trained with a dropout strategy to reduce the risk of overfitting. Then, outputs of the two branches are concentrated as a vector and input to the output layer. The detailed parameters of the RNNSurv are illustrated in Supplementary Table S3 after trial and error. RNNSurv was trained with an Adam optimizer, a learning rate of 0.05, and a training batch size of 64. Early termination was activated if the loss function did not decrease after 20 consecutive epochs; otherwise, the training process continued to 1000 epochs. The model was developed and internally validated by a 10-fold cross validation strategy on a development dataset, which consisted of data from patients admitted between 7 April 2016 and 17 March 2019. Simultaneously, back-elimination was employed to further improve performance and to reduce required predictors. In detail, the candidate predictor that had the greatest negative impact on testing performance (C-index) was deleted, and the process was repeated until no further predictor deletion would improve testing performance. After the model was developed, it was validated on an independent dataset, of which data were obtained in the same way as the development dataset but collected from patients admitted between 20 March 2019 and 16 March 2020. The detailed development and validation processes are shown in Figure 2.

In addition, a DeepSurv model [9] and a multivariate CPH model [6] were also developed and temporally validated for performance comparison. To avoid overfitting, the DeepSurv model employed a shallow structure with two FC layers. The first FC layer was followed by a ReLU activation function and batch normalization and trained using the dropout strategy as shown in Supplementary Figure S3. Its training configurations were the same as those of RNNSurv. The CPH model employed a penalty with 0.05 to the size of the coefficients during regression. This improved the stability of estimates and avoided convergence failure. The initial inputs, i.e., the candidate predictors obtained above, of the three models were the same. Because the DeepSurv and CPH models cannot capture temporal features by themselves, a covariate separately measured at admission and discharge would be regarded as two independent predictors. For example, heart rate (HR) was measured at both admission (HR_A) and discharge (HR_D), resulting in two predictors: HR_A and HR_D for DeepSurv and CPH. However, as RNNSurv could capture temporal information among time steps, it would regard the HR as one predictor but with two time steps (admission and discharge). After back-elimination, the optimal predictor sets may vary with models due to differences in algorithms.

### 2.5. Performance Evaluation

Discrimination performance of models was evaluated using Harrell’s C-index [23] and cumulative/dynamic time-dependent area under the receiver operating characteristic curve (AUC) [24]. Higher C-index and time-dependent AUC values indicate better discrimination. Calibration was evaluated at 1 and 2 years during follow-up using a calibration plot [25] and a Hosmer–Lemeshow test simultaneously. If the calibration plot lies on a 45-degree line, the calibration performance is perfect. Furthermore, the Brier score [26] was used for overall performance evaluation, where a score closer to 0 indicates better performance. Apart from the abovementioned measures, the models’ prediction performance was further evaluated in view of their risk stratification ability. The 20% of patients with lower predicted survival probabilities were considered at high risk of mortality [8]. Then, survival curves of high-risk and the other 80% patients (non-high-risk or low-risk) were checked using a Kaplan–Meier estimator and a log-rank test.

## 3. Results

### 3.1. Characteristics of Patients and Candidate Predictors

In total, 562 patients were included in the development dataset. The average age was 78 years and 45.7% of them were female. During follow-up of 30.9 ± 13.7 months, 81 (14.4%) patients died. Patients who died were older, with more prior hospital admissions, had a heavier burden on independence in daily life for the elderly with cognitive impairment (IDL) and activities of daily living (ADL), and were more likely to have chronic kidney disease (estimated glomerular filtration rate (eGFR) < 60 mL/min/1.73 m${}^{2}$), compared with patients who survived, as illustrated in Supplementary Table S1. The validation dataset included 168 patients of whom 28 (16.7%) patients died. Most characteristics were comparable between patients in the development and validation datasets, but patients in the validation dataset had lower body mass index (BMI), CHA${}_{2}$DS${}_{2}$-VAS${}_{c}$ score, and right ventricular systolic pressure (RVSP). The mortality between the development and validation datasets had no significant difference (14.4% vs. 16.7%, respectively, *p* = 0.551). The detailed comparison is illustrated in Supplementary Table S4.

After candidate predictor selection, age, length of stay, prior admission times, ischemic heart disease (IHD), New York Heart Association (NYHA) at discharge, frailty class, CHADS${}_{2}$, left ventricular ejection fraction (LVEF), mitral regurgitation (MR), tricuspid regurgitation (TR), RVSP, angiotensin-converting enzyme inhibitor/angiotensin receptor blocker (ACEi/ARB), NT-proB-type natriuretic peptide (NT-proBNP), hemoglobin (HGB), and time-varying covariates including eGFR, systolic blood pressure (SBP), diastolic blood pressure (DBP), HR, and low ADL were identified as initial candidate predictors for all-cause mortality, as illustrated in Supplementary Table S5.

### 3.2. Discrimination Evaluation

Candidate predictors were used to build initial models (RNNSurv, DeepSurv, and CPH). After back-elimination, the final predictors and C-index were obtained, as illustrated in Table 1. Models were internally validated by 10-fold cross validation and then temporally validated. Once the final used predictors were determined by back-elimination, models were trained on all of the data of the development dataset and then validated on the validation dataset. In 10-fold cross validation, the proposed RNNSurv achieved a 0.807 ± 0.057 (mean ± standard derivation) testing C-index on the development dataset and a C-index of 0.809 ± 0.022 on the validation dataset, whereas those of the DeepSurv model were 0.783 ± 0.066 and 0.762 ± 0.022, respectively, and those of the CPH model were 0.754 ± 0.069 and 0.764 ± 0.002, respectively. An ANOVA test with the Tukey method confirmed that the C-index of RNNSurv was significantly different from that of CPH and DeepSurv (RNNSurv vs. DeepSurv: *p* < 0.001; RNNSurv vs. CPH: *p* < 0.001). When RNNSurv, DeepSurv, and CPH were trained on all of the data of the development dataset, the validation C-indexes were 0.839, 0.755, and 0.764, respectively. In terms of predictors, the three models perform both generalizability and specificity. For example, all three models take age, length of stay, NT-proBNP, etc., as the predictors; while some predictors are model-specific, such as TR and IDL.

**Figure 3:**
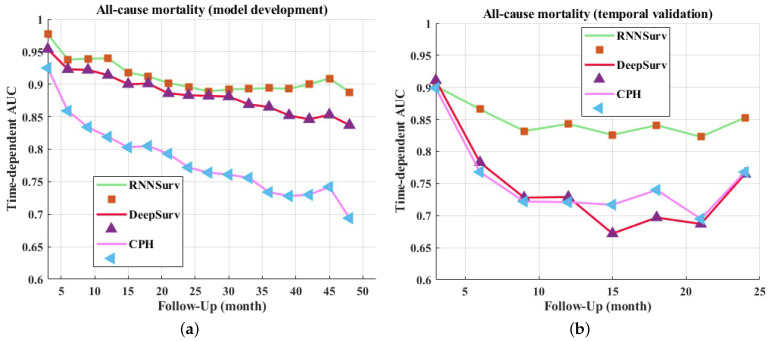
Time-dependent AUCs of models on development dataset (**a**) and validation dataset (**b**).

In addition to the C-index, the time-dependent AUCs of every 3 months are visualized in Figure 3. The upper boundary was set at 4 years for the development dataset and 2 years for the validation dataset to avoid high censoring rates at later follow-up times. The proposed model outperformed DeepSurv and CPH on both the development (Figure 3a) and validation datasets (Figure 3b).

### 3.3. Calibration Evaluation

**Figure 4:**
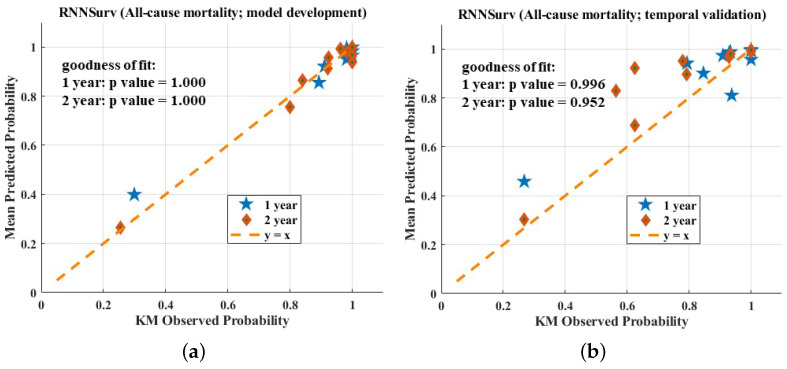

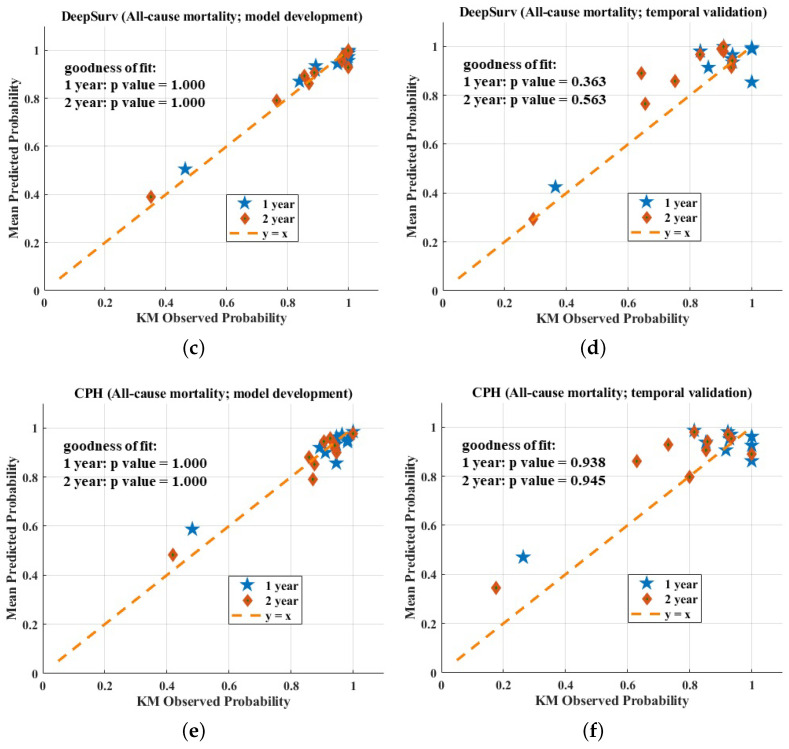
Calibration evaluation of RNNSurve (**a**,**b**), DeepSurve (**c**,**d**), and CPH model (**e**,**f**).

The calibration plots of the three models are shown in Figure 4. All three models fit well to a 45-degree line on the development dataset, indicating almost perfect calibration ability. On the validation dataset, the calibration plot of the proposed model seemed better than those of the other models. However, the three models showed a worse calibration ability on the validation dataset than the development dataset and the reason for this phenomenon has been explained by Park et al. [17]—if the sample size is small, few individuals will be included in subgroups. In this case, calibration analysis in view of the calibration plot and the statistical test are not robust or powerful enough.

### 3.4. Overall Evaluation

**Figure 5:**
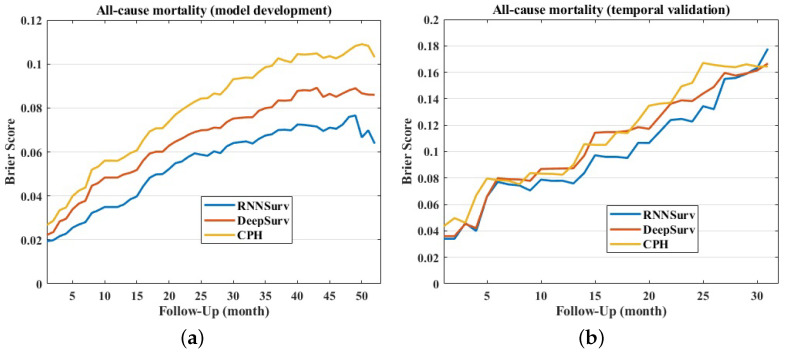
Brier scores of models on development dataset (**a**) and validation dataset (**b**).

The Brier score of the proposed model was smaller than those of DeepSurv and CPH on both the development and the validation datasets, as shown in Figure 5, indicating a better overall performance. In detail, the mean ± standard deviation of Brier scores of RNNSurv, DeepSurv, and CPH were 0.054 ± 0.017, 0.066 ± 0.020, and 0.080 ± 0.025 on the development dataset, and 0.099 ± 0.039, 0.107 ± 0.039, and 0.112 ± 0.040 on the validation dataset, respectively.

### 3.5. Risk Stratification

**Figure 6:**
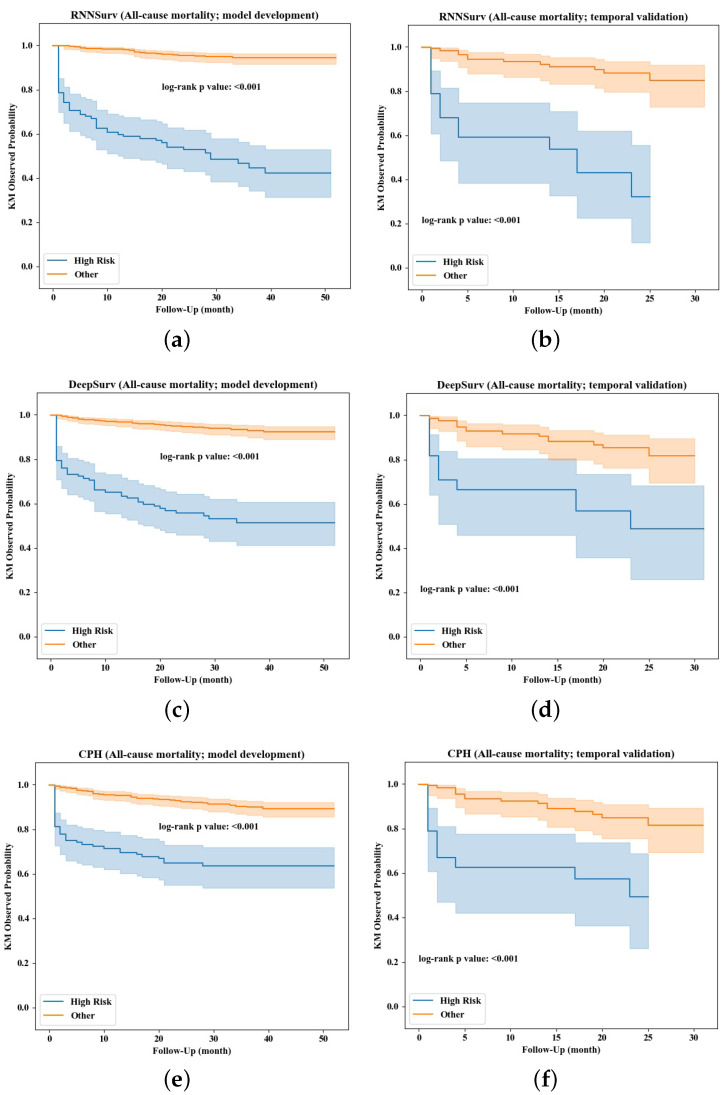
Risk stratification by the RNNSurve (**a**,**b**), DeepSurve (**c**,**d**), and CPH (**e**,**f**) models.

The prediction performance was further evaluated in view of the ability of risk stratification. Kaplan–Meier survival curves of high-risk and other (non-high-risk or low-risk) patients stratified by the three models are visualized in Figure 6. Compared with DeepSurv (Figure 6c,d) and CPH (Figure 6e,f), the survival curves of high-risk patients stratified by the proposed RNNSurv (Figure 6a,b) had more obvious differences from other patients on both the development and validation datasets, indicating a better ability of risk stratification. Mortality in the high-risk patient group and other or low-risk patient group stratified by RNNSurv had a significant difference (51.8% vs. 5.1%, *p* < 0.001 on the development dataset; 48.5% vs. 8.9%, *p* < 0.001 on the validation dataset). Patients with high risk of all-cause mortality were older, were more often female, had a heavier burden on their medical history in terms of IHD and vascular disease (VD), had a longer length of stay and severe HF symptoms assessed by NYHA and heavier burden on daily activities, and were more likely to have chronic kidney diseases compared with low-risk patients. Details are illustrated in Supplementary Table S6.

Additionally, significant risk and protective factors varied across all patients and high-risk patients only. Based on the results of univariate CPH analysis, in the patient group with a high risk of all-cause mortality, NYHA class (hazard ratio: 1.73 (95%CI 1.21–2.46), *p* = 0.002), low ADL (hazard ratio: 2.38 (95%CI 1.29–4.37), *p* = 0.005), creatinine (hazard ratio: 1.12 (95%CI 1.03–1.22), *p* = 0.011), and HR (hazard ratio: 1.02 (95%CI 1.00–1.03), *p* = 0.049) were significant risk factors, while protective factors were female (hazard ratio: 0.53 (95%CI 0.31–0.88), *p* = 0.015), direct oral anticoagulants/Warfarin usage (DOACWFuse) (hazard ratio: 0.47 (95%CI 0.27–0.83), *p* = 0.009), SBP (hazard ratio: 0.98 (95%CI 0.96–0.99), *p* < 0.001), and DBP (hazard ratio: 0.97 (95%CI 0.95–0.998), *p* = 0.031) (see Supplementary Table S7). However, in addition to the above significant factors, age (hazard ratio: 1.04 (95%CI 1.02–1.06), *p* < 0.001), CHA${}_{2}$DS${}_{2}$-VAS${}_{c}$ (hazard ratio: 1.15 (95%CI 1.02–1.30), *p* = 0.024), eGFR (hazard ratio: 0.98 (95%CI 0.96–0.99) at admission, *p* < 0.001; 0.96 (95%CI 0.94–0.97) at discharge, *p* < 0.001), etc., were also significantly associated with mortality in all patient groups (Supplementary Table S5).

## 4. Discussion

In this study, an RNN-based prognostic prediction model for patients with HF was developed and temporally validated. This model can consider time-varying covariates, which imply the course of disease (improving or worsening), and learn temporal information from these covariates for decision-making, while DeepSurv and CPH have no such ability. The proposed model achieved better prediction performance than DeepSurv and CPH in view of discrimination, calibration, and ability of risk stratification.

The multivariate CPH model is the most-used survival model to fit the relationship between patients’ covariates and outcomes [27]. However, its prediction ability may be limited by linear assumptions. Recently, Bazoukis et al. concluded that machine learning methods were important and effective for diagnosis, management, and prediction of outcomes in HF patients [28]. In addition, instead of expensive imaging measurement, Liu et al. reported a cheap method to estimate LVEF achieved by machine learning algorithms [29]. Coincidentally, a modern CPH feed-forward deep neural network model (DeepSurv) was proposed as an alternative method for survival or risk prediction [8,9]. The neural network-based model could learn and capture more complex and abstract features and feature combinations from input covariates by nonlinear activation functions in layers. Both CPH and DeepSurv assume that covariates or predictors are invariant. However, some covariates are time-varying due to treatments such as polypharmacy in real-world situations, which are informative and reflect the course of disease. These temporal features should be considered in prediction models. Conversely, the proposed RNN-based prediction model could “remember” previous information and use it for current decision-making; therefore, it naturally accepts and considers time-varying predictors by serially learning.

The proposed model identifies and uses predictors that are easily obtained by routine examination and are measured at admission and discharge. Most of these predictors are previously reported prognostic markers. Specifically, age, NYHA, LVEF, ACEi/ARB, HGB, and SBP were previously identified as predictors in the Seattle Heart Failure Model to predict 1-, 2-, and 3-year survival rates for patients with HF [30]. NT-proBNP was used as one of the predictors for clinical outcomes in patients with HFrEF [31]. Although eGFR was widely reported as strongly associated with outcome in HF, few studies used it as a predictor [32]. In our study, eGFR was identified as a predictor.

The proposed model has a considerable ability of risk stratification, especially in identifying patients who have high risk of mortality. Patients with high risk of all-cause mortality are much older. As reported by Gustafsson et al., elderly patients hospitalized with HF had a very critical prognosis [33]. NYHA classification is generally used to estimate HF symptoms, and it was reported to be a significant predictor of all-cause mortality [34]. In our study, NYHA was identified as a predictor of all-cause mortality as well, and a greater proportion of patients with high risk of all-cause mortality had high NYHA (e.g., NYHA III, IV). Shamagian et al. defined patients with eGFR < 30 mL/min/1.73 m${}^{2}$ as having severe renal failure, which was reported to be a strong predictor of mortality, and these patients had significantly poor survival rates [35]. In our study, eGFR was a significant predictor for all-cause mortality. The proportion of patients with eGFR < 30 mL/min/1.73 m${}^{2}$ in the high-risk group was about two or three times that of the non-high-risk group. It was reported that higher NT-proBNP levels were associated with higher incidences of all-cause mortality [36], which is consistent with our study. Dunlay et al. reported that mortality increased with increasing ADL difficulty [37] and low admission ADL was reported to be associated with cardiovascular mortality [38]. In this study, low ADL was a predictor for all-cause mortality and patients with high risk of all-cause mortality more often had low ADL at both admission and discharge. A systematic review and meta-analysis conducted by Lakhan et al. concluded that high C-reactive protein (CRP) was significantly associated with an increased risk of all-cause mortality and sometimes related to a greater risk of long-term adverse cardiovascular outcomes for patients with HF with preserved ejection fraction [39]. In this study with mixed cases, patients with high risk of all-cause mortality had significantly higher CRP than patients in the low-risk group.

Notably, significant risk and protective factors affecting survival or mortality are risk level-specific. Therefore, risk stratification or identifying patients at high risk are crucial to guide precision medicine and clinical strategies such as individually management and treatment. Based on the possible survival duration or probability of mortality after discharge, it is helpful in determining if or when the prevention treatment for high-risk patients should be introduced and determining if an early discharge could be conducted for low-risk patients to save costs and medical resources. Moreover, covariate value collected at discharge is more likely to perform a significant risk or protective effect on prognosis than the one collected at admission, indicating that covariates collected at discharge are more strongly associated with prognostic outcomes than ones collected at admission, and should be given more attention.

Limitations of the presented study are noted as follows. First, the proposed model was developed on a single-center dataset. Although a temporal validation, which is considered intermediate between internal and external validation, was conducted based on the TRIPOD report [13], an external validation is needed, such as conducting the validation on multiple centers and populations for further performance confirmation. Second, some predictors used in previously developed prediction models were not recorded, such as lymphocyte counts, uric acid, and sodium levels, which were used in the Seattle Heart Failure Model; therefore, performance comparison with these models is limited. Third, some covariates have missing data in varying proportions (>20%), limiting their inclusion in this study and prediction ability. Finally, the confirmation of event and survival information were determined by DPC data and medical records only, and thus it is possible that the event after discharge has not been fully confirmed or captured.

## 5. Conclusions

The proposed RNN-based risk prediction model for patients with HF demonstrated better performance than conventional statistical-based (CPH) and classical deep learning-based (DeepSurv) models. The model considers and accepts time-varying covariates, and in this way, it captures temporal information existing in a real-world situation. Risk and protective factors that are significantly associated with mortality are specific to risk levels, highlighting the demand for individual-specific clinical strategy instead of a uniform one-for-all strategy.

## Figures and Tables

**Table 1:** C-index and predictors of models.

Model	Model Development (10-Fold Cross Validation)	Validation	All of the Data	Predictors
RNNSurv	Train: 0.820 ± 0.022 Test: 0.807 ± 0.057	0.809 ± 0.022 (RNNSuev vs. DeepSurv: *p* value < 0.001; (RNNSuev vs. CPH: *p* value < 0.001;)	Development: 0.890 Validation: 0.839	Invariant predictors: Age, length of stay, IHD, NYHA at discharge, frailty, LVEF, TR, ACEi/ARB, NT-proBNP, HGB; Time-varying predictors: eGFR, SBP, DBP, HR, low ADL
DeepSurv	Train: 0.818 ± 0.033 Test: 0.783 ± 0.066	0.762 ± 0.022	Development: 0.872 Validation: 0.755	Age, length of stay, IHD, LVEF, RVSP, HGB, ACEi/ARB, IDL, NT-proBNP, (NYHA, eGFR, SBP, DBP, HR, low ADL) at discharge
CPH	Train: 0.768 ± 0.010 Test: 0.754 ± 0.069	0.764 ± 0.002	Development: 0.767 Validation: 0.762	Length of stay, IDL, ACEi/ARB, NT-proBNP, (NYHA, eGFR, SBP, low ADL) at discharge

## Data Availability

The data used in this article are not readily available because of the restrictions of the Institutional Review Board. Requests to access the datasets should be directed to the corresponding authors.

## Supplementary Materials

The following supporting information can be downloaded at: https://www.mdpi.com/article/10.3390/diagnostics12122947/s1, Table S1. Characteristics of Patients with Heart Failure for Model Development; Table S2. Comparison of Covariate Missing between Death and Survival Patient groups in Development Dataset; Table S3. Parameters of RNNSurv; Table S4. Comparison Between Patients in Development and Validation Datasets; Table S5. Candidate Predictors for All-cause Mortality; Table S6. Comparison Between High- and Low-Risk Patients Stratified by RNNSurv for All-Cause Mortality; Table S7. Univariate analysis for High-risk group (All-cause Mortality); Figure S1. Database construction flowchart; Figure S2. Structure of the proposed RNNSurv; Figure S3. Structure of DeepSurv.

## Abbreviations

The following abbreviations are used in this manuscript:

ACEi/ARB	Angiotensin-converting enzyme inhibitor/angiotensin receptor blocker
ADL	Activities of daily living
AUC	Area under receiver operating characteristic curve
BMI	Body mass index
CPH	Cox proportional hazard
CRP	C-reactive protein
DBP	Diastolic blood pressure
DPC	Diagnosis procedure combination
eGFR	Estimated glomerular filtration rate
FC	Fully connected
HF	Heart failure
HGB	Hemoglobin
HR	Heart rate
HR_A	HR measured at admission
HR_A	HR measured at discharge
IHD	Ischemic heart disease
IDL	Independence in daily life for the elderly with cognitive impairment
LVEF	Left ventricular ejection fraction
MR	Mitral regurgitation
NT-proBNP	NT-proB-type natriuretic peptide
NYHA	New York Heart Association
RNN	Recurrent neural network
RVSP	Right ventricular systolic pressure
SBP	Systolic blood pressure
TR	Tricuspid regurgitation
VD	Vfascular disease
