# Supplementary material for: Risk of Mortality Prediction Involving Time-Varying Covariates for Patients with Heart Failure Using Deep Learning
Source: Diagnostics (Basel). 2022 Nov 25;12(12):2947. doi: 10.3390/diagnostics12122947 (PMC9777280; doi:10.3390/diagnostics12122947)
Supplement: Supplementary file 1 [file diagnostics-12-02947-s001.zip › diagnostics-2046493-supplementary.pdf]

Supplementary Material

Supplementary Table S1. Characteristics of Patients with Heart Failure for Model Development

Covariates	Total (n=562)	All-cause mortality (n = 81)	Survive (n = 481)	P value
Follow Up (month)	30.9±13.7	9.9±10.3	34.4±10.8	<0.001
Cardiovascular mortality	39 (6.9)	39 (48.1)	0 (0)	NA
Age (years)	77.8±13.3	82.8±9.0	77.0±13.7	<0.001
Female	257 (45.7)	33 (40.7)	224 (46.6)	0.393
Length of stay (day)	19.3±15.7	19.3±13.2	19.3±16.1	0.467
Prior admission times	2.4±2.1	2.9±2.3	2.3±2.1	0.002
Weight (kg)	54.4±14.8	51.7±12.3	54.9±15.2	0.041
Body mass index (kg/m^2)	21.7±4.4	21.0±4.0	21.8±4.4	0.190
Hypertension	363 (64.6)	56 (69.1)	307 (63.8)	0.424
Hyperlipemia	252 (44.8)	42 (51.9)	210 (43.7)	0.211
Diabetes mellitus	190 (33.8)	27 (33.3)	163 (33.9)	0.977
Chronotropic incompetence	88 (15.7)	14 (17.3)	74 (15.4)	0.787
Ischemic heart disease	179 (31.9)	34 (42.0)	145 (30.2)	0.047
Peripheral arterial disease	50 (8.9)	10 (12.4)	40 (8.3)	0.333
Atrial fibrillation	303 (53.9)	43 (53.1)	260 (54.1)	0.967
Vascular disease	194 (34.5)	34 (42.0)	160 (33.3)	0.162
New York Heart Association (NYHA) at admission				0.469
1	24 (4.3)	4 (4.9)	20 (4.2)	
2	147 (26.2)	20 (24.7)	127 (26.4)	
3	281 (50.0)	37 (45.7)	244 (50.7)	
4	110 (19.6)	20 (24.7)	90 (18.7)	
NYHA at discharge				<0.001
1	66 (11.7)	5 (6.2)	61 (12.7)	
2	426 (75.8)	48 (59.3)	378 (78.6)	
3	54 (9.6)	18 (22.2)	36 (7.5)	
4	16 (2.9)	10 (12.4)	6 (1.3)	
Frail (frailty class≥3)	525 (93.4)	79 (97.5)	446 (92.7)	0.170
$CHADS_2$	3.0±1.2	3.2±1.2	2.9±1.2	0.044
CHA_2DS_2-VAS_C	5.2±1.8	5.6±1.7	5.1±1.8	0.034
Independence in daily life for the elderly with cognitive impairment				<0.001
0	425 (75.6)	48 (59.3)	377 (78.4)	
1	99 (17.6)	23 (28.4)	76 (15.8)	
2	31 (5.5)	9 (11.1)	22 (4.6)	
3	3 (0.5)	0 (0)	3 (0.6)	
4	4 (0.7)	1 (1.2)	3 (0.6)	
Low ADL at admission	72 (12.8)	17 (21.0)	55 (11.4)	0.028
Low ADL at discharge	31 (5.5)	14 (17.3)	17 (3.5)	<0.001

Left ventricular (LV) ejection fraction (%)	47.2±18.8	46.5±19.0	47.4±18.8	0.700
HFrEF	197 (35.0)	29 (35.8)	168 (34.9)	
HFmrEF	114 (20.3)	18 (22.2)	96 (20.0)	
HFpEF	251 (44.7)	34 (42.0)	217 (45.1)	
LV end-diastolic volume (mL)	124.8±58.7	131.4±73.7	123.7±55.9	0.845
LV end-diastolic dimension (mm)	49.7±10.2	50.5±12.2	49.6±9.8	0.506
LV end-systolic diameter (mm)	38.1±12.3	39.2±14.3	37.9±12.0	0.678
Left atrial diameter (mm)	45.0±8.4	46.0±8.8	44.9±8.3	0.303
Mitral regurgitation	1.5±1.1	1.8±1.1	1.5±1.1	0.030
Tricuspid regurgitation	1.3±1.1	1.6±1.2	1.3±1.0	0.034
RVSP (mmHg)	29.2±10.3	31.0±11.1	28.9±10.2	0.053
Atrial septal defect after ablation	30 (5.3)	4 (4.9)	26 (5.4)	0.925
Aortic valve replacement	27 (4.8)	5 (6.2)	22 (4.6)	0.733
Hemodialysis	21 (3.7)	5 (6.2)	16 (3.3)	0.351
ICD	5 (0.9)	0 (0)	5 (1.0)	1
CRT-D	24 (4.3)	5 (6.2)	19 (4.0)	0.370
CRT-P	4 (0.7)	1 (1.2)	3 (0.6)	0.464
Pacemaker	66 (11.7)	10 (12.3)	56 (11.6)	1
ACEi/ARB	268 (47.7)	51 (63.0)	217 (45.1)	0.004
Beta blockers	286 (50.9)	48 (59.3)	238 (49.5)	0.131
Mineralocorticoid receptor antagonist	149 (26.5)	28 (34.6)	121 (25.2)	0.101
Diuretic	302 (53.7)	49 (60.5)	253 (52.6)	0.231
DOACWFuse at admission	208 (37.0)	34 (42.0)	174 (36.2)	0.381
DOACWFuse at discharge	272 (48.4)	34 (42.0)	238 (49.5)	0.919
eGFR at admission (mL/min/1.73m^2)	42.8±21.1	34.7±21.4	44.2±20.8	<0.001
< 60 mL/min/1.73m^2	460 (81.9)	70 (86.4)	390 (81.1)	
eGFR at discharge (mL/min/1.73m^2)	40.8±21.2	29.2±18.9	42.7±21.0	<0.001
< 60 mL/min/1.73m^2	494 (87.9)	77 (95.1)	417 (86.7)	
Creatinine clearance rate (CrC) at admission (mL/min)	40.3±26.9	28.0±17.6	42.4±27.6	<0.001
CrC at discharge (mL/min)	37.9±24.8	24.6±16.4	40.1±25.3	<0.001

Creatinine (Cr) at admission (mg/dL)	1.5±1.3	1.9±1.5	1.4±1.2	<0.001
Cr at discharge (mg/dL)	1.6±1.5	2.5±2.5	1.4±1.2	<0.001
Log(NT-proB-type natriuretic peptide) (pg/mL)	3.6±0.5	3.9±0.5	3.6±0.5	<0.001
Albumin (g/dL)	3.4±0.5	3.3±0.5	3.4±0.5	0.007
Total protein (g/dL)	7.0±0.6	7.0±0.6	7.0±0.6	0.592
C-reactive protein (mg/dL)	2.3±4.1	2.6±3.6	2.3±4.2	0.041
Hemoglobin (g/dL)	11.9±2.4	11.3±2.1	12.0±2.4	0.008
Systolic blood pressure (SBP) at admission (mmHg)	132.1±25.9	128.2±31.9	132.7±24.7	0.224
SBP at discharge (mmHg)	112.2±18.1	104.9±24.7	113.4±16.4	0.003
Diastolic blood pressure (DBP) at admission (mmHg)	72.8±32.2	76.9±73.4	72.1±17.7	0.272
DBP at discharge (mmHg)	59.3±10.3	55.9±12.8	59.9±9.7	0.008
Heart rate at admission (bmp)	85.3±23.1	83.5±19.4	85.6±23.7	0.394
Heart rate at discharge (bmp)	70.2±13.9	73.2±18.6	69.7±12.9	0.104

ACEi (Angiotensin-converting enzyme inhibitor); ADL (activities of daily living); ARB (Angiotensin receptor blocker); $CHADS_2$ (congestive heart failure: 1 point; hypertension: 1 point, age ≥ 75 years: 1 point; diabetes mellitus: 1 point; prior stroke or TIA or thromboembolism: 2 points); $CHAD_2DS_2\text{-}VAS_C$ score (congestive heart failure: 1 point, hypertension: 1 point; age ≥ 75 years: 2 points; diabetes mellitus: 1 point; prior stroke or TIA or thromboembolism: 2 points; vascular disease: 1 point; age 65-74 years: 1 point; sex category: 1 point); CRT-D (cardiac resynchronization therapy defibrillator); CRT-P (cardiac resynchronization therapy pacemaker); DOACWFuse (Direct oral anticoagulants or Warfarin usage); eGFR (estimated glomerular filtration rate); HFmrEF (heart failure with mid-range ejection fraction); HFpEF (heart failure with preserved ejection fraction); HFrEF (heart failure with reduced ejection fraction); ICD (implantable cardioverter defibrillator); NYHA (New York Heart Association); RVSP (Right ventricular systolic pressure)

Supplementary Table S2. Comparison of Covariate Missing between Death and Survival Patient groups in Development Dataset

Covariates	All-cause mortality (n = 81)	Survive (n = 481)	Abs(difference) (%)
Age (years)	0 (0)	0 (0)	0
Female	0 (0)	0 (0)	0
Length of stay (day)	0 (0)	0 (0)	0
Weight (kg)	1 (1.2)	4 (0.8)	0.4
Hypertension	0 (0)	0 (0)	0
Hyperlipemia	0 (0)	0 (0)	0
Diabetes mellitus	0 (0)	0 (0)	0
Chronotropic incompetence	0 (0)	0 (0)	0
Ischemic heart disease	0 (0)	1 (0.2)	0.2
Peripheral arterial disease	0 (0)	0 (0)	0
Atrial fibrillation	0 (0)	0 (0)	0
Vascular disease	0 (0)	1 (0.2)	0.2
New York Heart Association (NYHA) at admission	0 (0)	0 (0)	0
NYHA at discharge	0 (0)	0 (0)	0
Frail	0 (0)	4 (0.8)	0.8
$CHADS_2$	0 (0)	0 (0)	0
CHA_2DS_2-VAS_C	0 (0)	1 (0.2)	0.2
Independence in daily life for the elderly with cognitive impairment	3 (3.7)	50 (10.4)	6.7
Low activities of daily life (ADL) at admission	1 (1.2)	3 (0.6)	0.6
Low ADL at discharge	6 (7.4)	20 (4.2)	3.2
Left ventricular (LV) ejection fraction (%)	6 (7.4)	35 (7.3)	0.1
LV end-diastolic volume (mL)	8 (9.9)	45 (9.4)	0.5
LV end-diastolic dimension (mm)	6 (7.4)	35 (7.3)	0.1
LV end-systolic diameter (mm)	6 (7.4)	36 (7.5)	0.1
Left atrial diameter (mm)	7 (8.6)	42 (8.7)	0.1
Mitral regurgitation	6 (7.4)	37 (7.7)	0.3
Tricuspid regurgitation	8 (9.9)	39 (8.1)	1.1
Right ventricular systolic pressure (mmHg)	16 (19.8)	95 (19.8)	0
Atrial septal defect after ablation	0 (0)	0 (0)	0
Aortic valve replacement	0 (0)	0 (0)	0
Hemodialysis	0 (0)	0 (0)	0
Angiotensin-converting enzyme inhibitor/Angiotensin receptor blocker	2 (2.5)	3 (0.6)	1.9
Beta blockers	2 (2.5)	2 (0.4)	2.1
Diuretic	2 (2.5)	3 (0.6)	1.9
Direct oral anticoagulants/Warfarin usage at admission	1 (1.2)	2 (0.4)	0.8

Direct oral anticoagulants/Warfarin usage at discharge	4 (4.9)	5 (1.0)	3.9
Estimated glomerular filtration rate (eGFR) at admission (mL/min/1.73m^2)	0 (0)	3 (0.6)	0.6
eGFR at discharge (mL/min/1.73m^2)	1 (1.2)	31 (6.4)	5.2
Creatinine clearance rate (CrC) at admission (mL/min)	2 (2.5)	6 (1.2)	1.3
CrC at discharge (mL/min)	3 (3.7)	34 (7.1)	3.4
Creatinine (Cr) at admission (mg/dL)	0 (0)	3 (0.6)	0.6
Cr at discharge (mg/dL)	1 (1.2)	31 (6.4)	5.2
Log(NT-proB-type natriuretic peptide)(pg/mL)	10 (12.3)	44 (9.1)	3.2
Albumin (g/dL)	6 (7.4)	39 (8.1)	0.7
Total protein (g/dL)	7 (8.6)	40 (8.3)	0.3
C-reactive protein (mg/dL)	1 (1.2)	11 (2.3)	1.1
Hemoglobin (g/dL)	0 (0)	4 (0.8)	0.8
Systolic blood pressure (SBP) at admission (mmHg)	0 (0)	0 (0)	0
SBP at discharge (mmHg)	0 (0)	5 (1.0)	1
Diastolic blood pressure (DBP) at admission (mmHg)	0 (0)	0 (0)	0
DBP at discharge (mmHg)	0 (0)	5 (1.0)	1
Heart rate at admission (bmp)	0 (0)	1 (0.2)	0.2
Heart rate at discharge (bmp)	0 (0)	5 (1.0)	1

Supplementary Table S3. Parameters of RNNSurv

<table>
<tr><td>Time-varying predictors
(m1-dimension; 2-time steps)</td><td>Invariant predictors
(m2-dimension)</td></tr>
<tr><td>Recurrent neural network (RNN) layer
(input_size = m1, hidden_size = m1,
num_layers = 1, batch_first = True)</td><td rowspan="2">Fully connected (FC) layer
(in_features = m2, out_features = 16)
ReLu activation function,
BatchNorm1d(num_features = 16)
Droup(p = 0.5)</td></tr>
<tr><td>FC layer(in_features = m1, out_features = m1)
ReLu activation function,
BatchNorm1d(num_features = m1)
Droup(p = 0.5)</td></tr>
<tr><td colspan="2" align="center">Concentrate</td></tr>
<tr><td colspan="2" align="center">FC layer(in_features = m1 + 16, out_features = 1)
Linear activation function</td></tr>
</table>

Due to back elimination, the number of predictors (m1, m2) would change, therefore, the parameter values in RNNSurv would change as well. Then the model would be fixed once back elimination was done. Model was developed by PyTorch 1.5.0 (Curran Associates, Inc.).

Supplementary Table S4. Comparison Between Patients in Development and Validation Datasets

Covariates	Development (n=562)	Validation (n=168)	P value
Follow Up (month)	30.9±13.7	13.6±10.2	<0.001
All-cause mortality	81 (14.4)	28 (16.7)	0.551
Cardiovascular mortality	39 (6.9)	17 (10.1)	0.233
Age (years)	77.8±13.3	78.0±12.8	0.927
Female	257 (45.7)	74 (44.1)	0.767
Length of stay (day)	19.3±15.7	18.5±13.3	0.836
Weight (kg)	54.4±14.8	52.8±12.0	0.142
Body mass index (kg/m^2)	21.7±4.4	20.8±3.7	0.017
Hypertension	363 (64.6)	107 (63.7)	0.903
Hyperlipemia	252 (44.8)	73 (43.5)	0.819
Diabetes mellitus	190 (33.8)	53 (31.6)	0.651
Chronotropic incompetence	88 (15.7)	32 (19.1)	0.357
Ischemic heart disease	179 (31.9)	64 (38.1)	0.157
Peripheral arterial disease	50 (8.9)	22 (13.1)	0.146
Atrial fibrillation	303 (53.9)	94 (56.0)	0.706
Vascular disease	194 (34.5)	74 (44.1)	0.031
New York Heart Association (NYHA) at admission			0.080
1	24 (4.3)	17 (10.1)	
2	147 (26.2)	42 (25.0)	
3	281 (50.0)	83 (49.4)	
4	110 (19.6)	26 (15.5)	
NYHA at discharge			0.005
1	66 (11.7)	38 (22.6)	
2	426 (75.8)	112 (66.7)	
3	54 (9.6)	12 (7.1)	
4	16 (2.9)	6 (3.6)	
Frail (frailty class≥3)	525 (93.4)	151 (89.9)	0.171
$CHADS_2$	3.0±1.2	3.0±1.2	0.800
CHA_2DS_2-VAS_C	5.2±1.8	4.7±1.6	0.002
Independence in daily life for the elderly with cognitive impairment			0.627
0	425 (75.6)	134 (79.8)	
1	99 (17.6)	11 (6.6)	
2	31 (5.5)	10 (6.0)	
3	3 (0.5)	7 (4.2)	
4	4 (0.7)	6 (3.6)	
Low activities of daily life (ADL) at admission	72 (12.8)	18 (10.7)	0.554
Low ADL at discharge	31 (5.5)	8 (4.8)	0.853
Left ventricular (LV) ejection fraction (%)	47.2±18.8	45.1±18.6	0.192
HFrEF	197 (35.0)	67 (39.9)	
HFmrEF	114 (20.3)	30 (17.8)	
HFpEF	251 (44.7)	71 (42.3)	

LV end-diastolic volume (mL)	124.8±58.7	121.0±50.3	0.715
LV end-diastolic dimension (mm)	49.7±10.2	49.1±9.0	0.433
LV end-systolic diameter (mm)	38.1±12.3	38.1±11.2	0.777
Left atrial diameter (mm)	45.0±8.4	44.3±8.6	0.356
Mitral regurgitation	1.5±1.1	1.7±1.0	0.014
Tricuspid regurgitation	1.3±1.1	1.3±1.0	0.365
Right ventricular systolic pressure (mmHg)	29.2±10.3	27.3±10.2	0.034
Atrial septal defect after ablation	30 (5.3)	13 (7.7)	0.331
Aortic valve replacement	27 (4.8)	10 (6.0)	0.693
Hemodialysis	21 (3.7)	5 (3.0)	0.819
Angiotensin-converting enzyme inhibitor/Angiotensin receptor blocker	268 (47.7)	64 (38.1)	0.036
Beta blockers	286 (50.9)	85 (50.6)	1
Mineralocorticoid receptor antagonist	149 (26.5)	37 (22.0)	0.284
Diuretic	302 (53.7)	73 (43.5)	0.024
Direct oral anticoagulants/Warfarin usage at admission	208 (37.0)	73 (43.5)	0.157
Direct oral anticoagulants/Warfarin usage at discharge	272 (48.4)	90 (53.6)	0.276
Estimated glomerular filtration rate (eGFR) at admission (mL/min/1.73m^2)	42.8±21.1	44.1±19.6	0.458
eGFR at discharge (mL/min/1.73m^2)	40.8±21.2	47.4±61.3	0.197
Creatinine clearance rate (CrC) at admission (mL/min)	40.3±26.9	36.6±20.2	0.382
CrC at discharge (mL/min)	37.9±24.8	38.1±34.8	0.669
Creatinine (Cr) at admission (mg/dL)	1.5±1.3	1.4±1.0	0.257
Cr at discharge (mg/dL)	1.6±1.5	1.5±1.3	0.063
Log(NT-proB-type natriuretic peptide)(pg/mL)	3.6±0.5	3.6±0.6	0.808
Albumin (g/dL)	3.4±0.5	3.4±0.5	0.491
Total protein (g/dL)	7.0±0.6	6.9±0.7	0.094
C-reactive protein (mg/dL)	2.3±4.1	2.1±4.2	0.121
Hemoglobin (g/dL)	11.9±2.4	12.1±2.3	0.316
Systolic blood pressure (SBP) at admission (mmHg)	132.1±25.9	139.0±32.8	0.066
SBP at discharge (mmHg)	112.2±18.1	119.8±81.0	0.422
Diastolic blood pressure (DBP) at admission (mmHg)	72.8±32.2	80.6±24.1	<0.001
DBP at discharge (mmHg)	59.3±10.3	61.5±10.4	0.017
Heart rate at admission (bmp)	85.3±23.1	91.7±27.3	0.006
Heart rate at discharge (bmp)	70.2±13.9	70.9±14.6	0.592

Supplementary Table S5. Candidate Predictors for All-cause Mortality

Univariate CPH analysis			LASSO analysis	Candidate predictors after multicollinearity checking
Selected candidate covariates (P < 0.05)	Hazard ratio (95% confidence interval)	P value	Selected candidate covariates (Coefficient ≠ 0)	
Age	1.04 (1.02-1.06)	<0.001	Age	Age; Length of stay; Prior admission times; IHD; NYHA at discharge; Frailty; $CHADS_2$; LVEF; MR; TR; RVSP; ACEi/ARB; NT-proBNP; HGB; IDL; [eGFR; SBP; DBP; HR; ADL_low] at admission and discharge
Prior admission times	1.10 (1.02-1.19)	0.016	Length of stay	
Ischemic heart disease (IHD)	1.60 (1.03-2.48)	0.038	Left ventricular (LV) ejection fraction (LVEF)	
New York Heart Association (NYHA) at discharge	3.22 (2.39-4.34)	<0.001	LV end-diastolic volume (LVEDV)	
Frailty	1.37 (1.17-1.59)	<0.001	Right ventricular systolic pressure (RVSP)	
$CHADS_2$	1.20 (1.003-1.43)	0.046	eGFR at admission	
CHA_2DS_2-VAS_C	1.15 (1.02-1.30)	0.024	eGFR at discharge	
Mitral regurgitation (MR)	1.22 (1.01-1.48)	0.044	CrC at admission	
Tricuspid regurgitation (TR)	1.26 (1.05-1.52)	0.015	CrC at discharge	
Angiotensin-converting enzyme inhibitor/Angiotensin receptor blocker (ACEi/ARB)	1.92 (1.22-3.01)	0.005	SBP at admission	
Estimated glomerular filtration rate (eGFR) at admission	0.98 (0.96-0.99)	<0.001	SBP at discharge	
eGFR at discharge	0.96 (0.94-0.97)	<0.001	DBP at admission	
Creatinine clearance rate (CrC) at admission	0.97 (0.96-0.98)	<0.001	DBP at discharge	
CrC at discharge	0.96 (0.94-0.97)	<0.001	HR at admission	
Diastolic blood pressure (DBP) at admission	1.01 (1.001-1.01)	0.015	HR at discharge	
Systolic blood pressure (SBP) at discharge	0.97 (0.95-0.98)	<0.001	Cr at discharge	
DBP at discharge	0.96 (0.94-0.98)	<0.001	NT-proBNP	
Heart rate (HR) at discharge	1.02 (1.003-1.03)	0.023		
Creatinine (Cr) at admission	1.20 (1.08-1.33)	<0.001		
Cr at discharge	1.28 (1.19-1.38)	<0.001		

NT-proB-type natriuretic peptide (NT-proBNP)	1.00 (1.00-1.00)	0.003		
Hemoglobin (HGB)	0.89 (0.81-0.97)	0.008		
Independence in daily life for the elderly with cognitive impairment (IDL)	1.53 (1.21-1.95)	<0.001		
Low activities of daily life (ADL) at admission	2.14 (1.25-3.66)	0.005		
Low ADL at discharge	5.82 (3.25-10.41)	<0.001		

Supplementary Table S6. Comparison Between High- and Low-Risk Patients Stratified by RNNSurv for All-Cause Mortality

Covariates	High risk (n=112)	Low risk (n=450)	P value
Follow Up (month)	19.6±15.9	33.7±11.6	<0.001
All-cause mortality	58 (51.8)	23 (5.1)	<0.001
Age (years)	83.2±9.1	76.5±13.8	<0.001
Female	63 (56.3)	194 (43.1)	0.017
Length of stay (day)	20.8±13.2	18.9±16.3	0.006
Weight (kg)	50.3±11.4	55.5±15.4	<0.003
Body mass index (kg/m^2)	21.1±3.9	21.9±4.5	0.167
Hypertension	72 (64.3)	291 (64.7)	1
Hyperlipemia	55 (49.1)	197 (43.8)	0.364
Diabetes mellitus	35 (31.3)	155 (34.4)	0.598
Chronotropic incompetence	21 (18.8)	67 (14.9)	0.389
Ischemic heart disease	51 (45.5)	128 (28.4)	<0.001
Peripheral arterial disease	15 (13.4)	35 (7.8)	0.093
Atrial fibrillation	63 (56.3)	240 (53.3)	0.654
Vascular disease	53 (47.3)	141 (31.3)	0.002
New York Heart Association (NYHA) at admission			<0.001
1	3 (2.7)	21 (4.7)	
2	18 (16.1)	129 (28.7)	
3	60 (53.6)	221 (49.1)	
4	31 (27.7)	79 (17.6)	
NYHA at discharge			<0.001
1	4 (3.6)	62 (13.8)	
2	62 (55.4)	364 (80.9)	
3	33 (29.5)	21 (4.7)	
4	13 (11.6)	3 (0.7)	
Frail (frailty class≥3)	111 (99.1)	414 (92.0)	0.012
$CHADS_2$	3.2±1.1	2.9±1.2	0.064
CHA_2DS_2-VASc	5.8±1.7	5.0±1.8	<0.001
Independence in daily life for the elderly with cognitive impairment			<0.001
0	67 (59.8)	358 (79.6)	
1	28 (25.0)	71 (15.8)	
2	13 (11.6)	18 (4.0)	
3	1 (0.9)	2 (0.4)	
4	3 (2.7)	1 (0.2)	
Low activities of daily life (ADL) at admission	30 (26.8)	42 (9.3)	<0.001
Low ADL at discharge	21 (18.8)	10 (2.2)	<0.001
Left ventricular (LV) ejection fraction (%)	47.1±19.0	47.3±18.7	0.976
LV end-diastolic volume (mL)	124.4±62.2	124.9±57.9	0.895
LV end-diastolic dimension (mm)	49.2±11.1	49.8±10.0	0.602
LV end-systolic diameter (mm)	37.8±13.0	38.1±12.2	0.782
Left atrial diameter (mm)	46.2±8.7	44.7±8.3	0.121

Mitral regurgitation	1.9±1.2	1.4±1.0	<0.001
Tricuspid regurgitation	1.7±1.3	1.2±1.0	<0.001
Right ventricular systolic pressure (mmHg)	32.0±11.4	28.4±9.9	0.002
Atrial septal defect after ablation	6 (5.4)	24 (5.3)	1
Aortic valve replacement	4 (3.6)	23 (5.1)	0.664
Hemodialysis	10 (8.9)	11 (2.4)	0.003
Angiotensin-converting enzyme inhibitor/Angiotensin receptor blocker	67 (59.8)	201 (44.7)	0.006
Beta blockers	63 (56.3)	223 (49.6)	0.245
Mineralocorticoid receptor antagonist	37 (33.0)	112 (24.9)	0.104
Diuretic	65 (58.0)	237 (52.7)	0.361
Direct oral anticoagulants/Warfarin usage at admission	42 (37.5)	166 (36.9)	0.992
Direct oral anticoagulants/Warfarin usage at discharge	43 (38.4)	229 (50.9)	0.024
Estimated glomerular filtration rate (eGFR) at admission (mL/min/1.73m^2)	33.6±23.2	45.1±20.0	<0.001
eGFR at admission > 60 mL/min/1.73m^2	12 (10.7)	90 (20.0)	
30≤eGFR at admission≤60 mL/min/1.73m^2	39 (34.8)	258 (57.3)	
eGFR at admission < 30 mL/min/1.73m^2	61 (54.5)	102 (22.7)	
eGFR at discharge (mL/min/1.73m^2)	29.1±21.9	43.7±20.1	<0.001
eGFR at discharge > 60 mL/min/1.73m^2	7 (6.3)	61 (13.6)	
30≤eGFR at discharge≤60 mL/min/1.73m^2	36 (32.1)	284 (63.1)	
eGFR at discharge < 30 mL/min/1.73m^2	69 (61.6)	105 (23.3)	
Creatinine clearance rate (CrC) at admission (mL/min)	24.8±15.9	44.2±27.7	<0.001
CrC at discharge (mL/min)	22.4±14.9	41.7±25.3	<0.001
Creatinine (Cr) at admission (mg/dL)	2.0±1.7	1.3±1.1	<0.001
Cr at discharge (mg/dL)	2.5±2.5	1.4±1.0	<0.001
Log(NT-proB-type natriuretic peptide) (pg/mL)	4.0±0.5	3.6±0.5	<0.001
Albumin (g/dL)	3.2±0.5	3.5±0.5	<0.001
Total protein (g/dL)	7.0±0.7	7.0±0.6	0.815
C-reactive protein (mg/dL)	2.8±4.0	2.2±4.1	0.005
Hemoglobin (g/dL)	11.2±2.1	12.1±2.4	<0.001
Systolic blood pressure (SBP) at admission (mmHg)	126.7±32.1	133.4±23.9	0.041
SBP at discharge (mmHg)	105.0±23.7	114.0±15.9	<0.001
Diastolic blood pressure (DBP) at admission (mmHg)	75.6±63.4	72.1±17.4	0.088
DBP at discharge (mmHg)	56.3±12.2	60.1±9.6	0.003
Heart rate at admission (bmp)	85.0±23.3	85.3±23.1	0.897
Heart rate at discharge (bmp)	73.1±16.9	69.5±12.9	0.039

Supplementary Table S7. Univariate analysis for High-risk group (All-cause Mortality)

Covariate	Hazard ratio	P value
Female	0.53 (0.31-0.88)	0.015
NYHA at discharge	1.73 (1.21-2.46)	0.002
Low activities of daily life at discharge	2.38 (1.29-4.37)	0.005
Direct oral anticoagulants/Warfarin usage at discharge	0.47 (0.27-0.83)	0.009
Creatinine at discharge	1.12 (1.03-1.22)	0.011
Systolic blood pressure at discharge	0.98 (0.96-0.99)	<0.001
Diastolic blood pressure at discharge	0.97 (0.95-0.998)	0.031
Heart rate at discharge	1.02 (1.00-1.03)	0.049

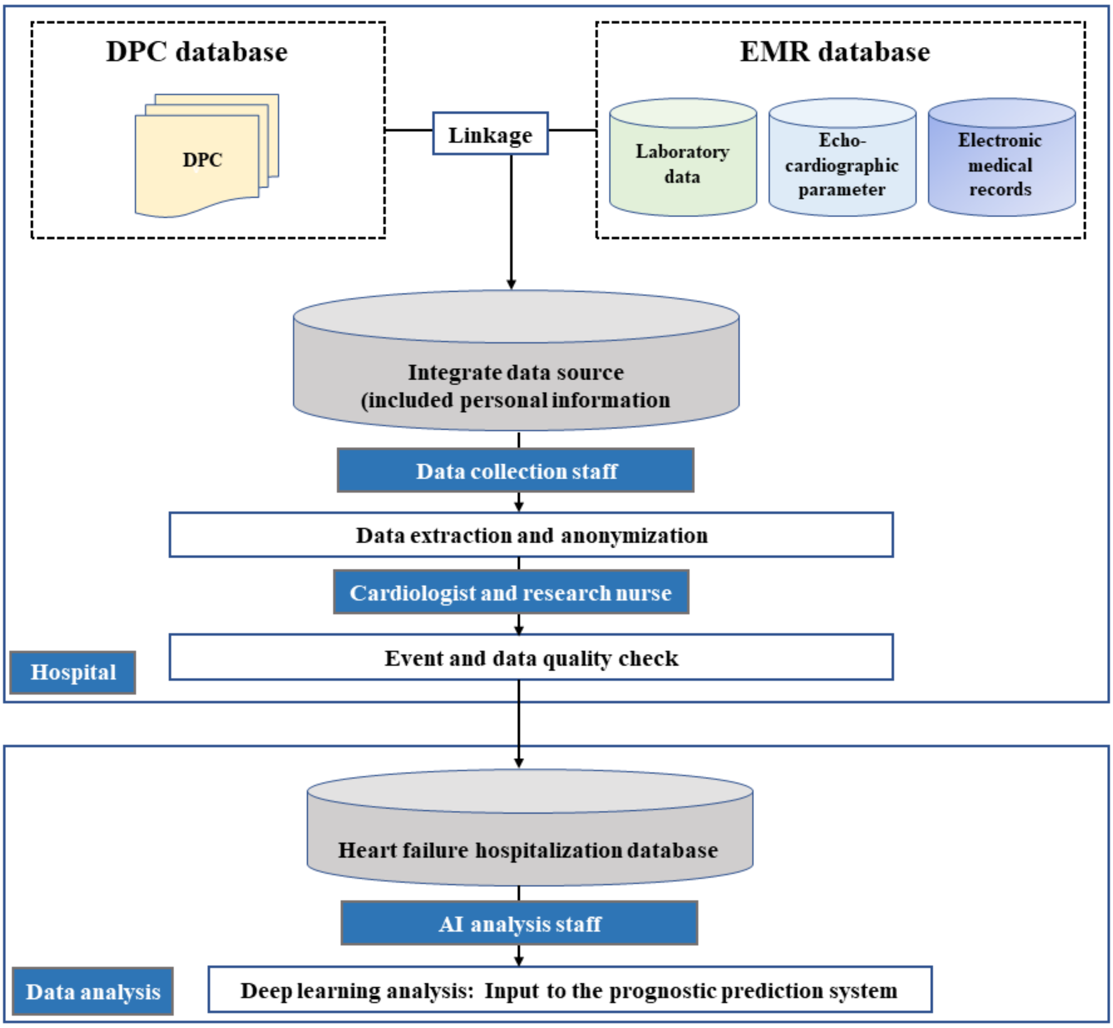

Supplementary Figure S1. Database construction flowchart.

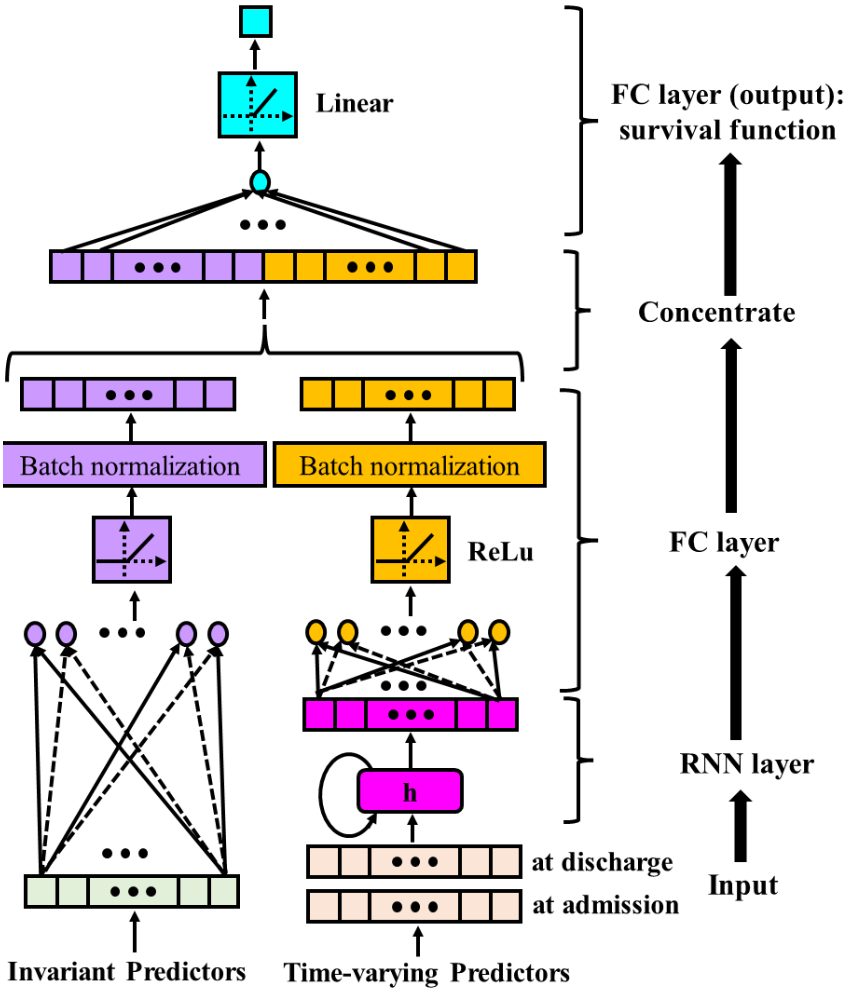

Supplementary Figure S2. Structure of the proposed RNNSurv.

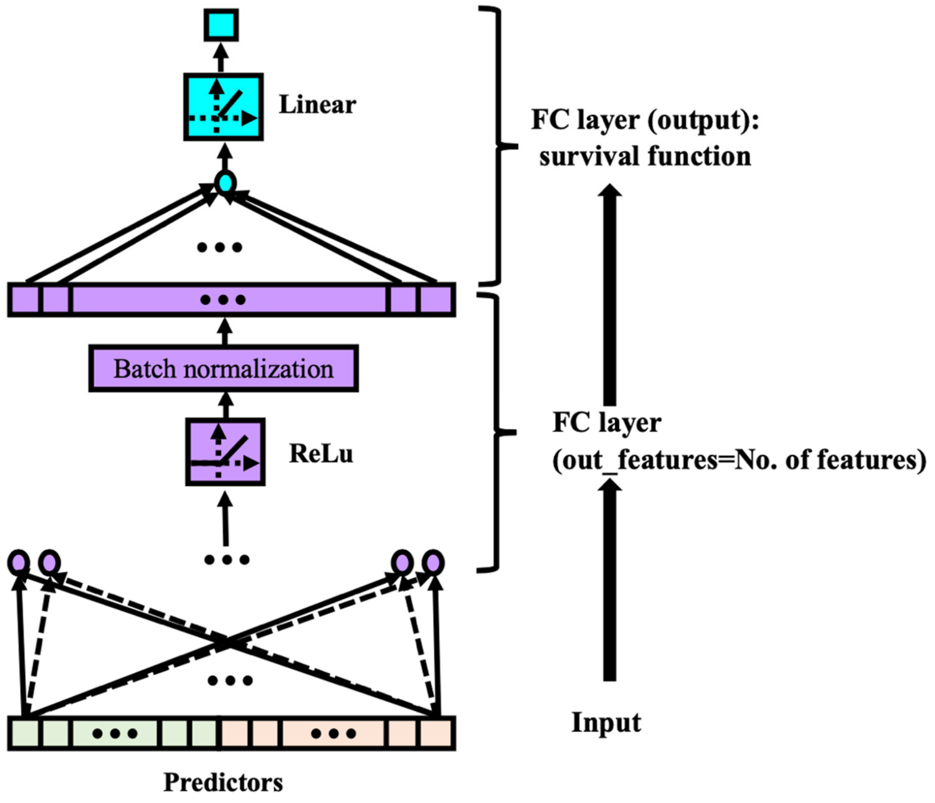

Supplementary Figure S3. Structure of DeepSurv.
